# Alternative Splicing of *Spg7*, a Gene Involved in Hereditary Spastic Paraplegia, Encodes a Variant of Paraplegin Targeted to the Endoplasmic Reticulum

**DOI:** 10.1371/journal.pone.0036337

**Published:** 2012-05-01

**Authors:** Giuseppe Mancuso, Esther Barth, Pietro Crivello, Elena I. Rugarli

**Affiliations:** 1 Institute of Zoology, University of Cologne, Köln, Germany; 2 National Neurological Institute Carlo Besta, Milan, Italy; 3 Cologne Excellence Cluster on Cellular Stress Responses in Aging-Associated Diseases (CECAD), University of Cologne, Cologne, Germany; 4 Center for Molecular Medicine (CMMC), University of Cologne, Cologne, Germany; Oregon Health and Science University, United States of America

## Abstract

**Background:**

Hereditary spastic paraplegia defines a group of genetically heterogeneous diseases characterized by weakness and spasticity of the lower limbs owing to retrograde degeneration of corticospinal axons. One autosomal recessive form of the disease is caused by mutation in the *SPG7* gene. Paraplegin, the product of *SPG7*, is a component of the *m*-AAA protease, a high molecular weight complex that resides in the mitochondrial inner membrane, and performs crucial quality control and biogenesis functions in mitochondria.

**Principal Findings:**

Here we show the existence in the mouse of a novel isoform of paraplegin, which we name paraplegin-2, encoded by alternative splicing of *Spg7* through usage of an alternative first exon. Paraplegin-2 lacks the mitochondrial targeting sequence, and is identical to the mature mitochondrial protein. Remarkably, paraplegin-2 is targeted to the endoplasmic reticulum. We find that paraplegin-2 exposes the catalytic domains to the lumen of the endoplasmic reticulum. Moreover, endogenous paraplegin-2 accumulates in microsomal fractions prepared from mouse brain and retina. Finally, we show that the previously generated mouse model of *Spg7-*linked hereditary spastic paraplegia is an isoform-specific knock-out, in which mitochondrial paraplegin is specifically ablated, while expression of paraplegin-2 is retained.

**Conclusions/Significance:**

These data suggest a possible additional role of AAA proteases outside mitochondria and open the question of their implication in neurodegeneration.

## Introduction

Hereditary Spastic Paraplegias (HSPs) are genetically heterogeneous diseases characterized by progressive spasticity and weakness of the lower limbs owing to retrograde degeneration of cortical motor neuron axons [1]. The age at onset is variable, ranging from early childhood to 70 years of age. More than 20 proteins involved in HSP have been so far identified, and their functional studies begin to shed light on pathogenic pathways [2]. The identification of HSP patients carrying mutations in the mitochondrial protease paraplegin and in the chaperone HSP60 has established disturbances of mitochondrial protein quality control pathways as one of the pathogenic mechanisms in this disease [3], [4].

Paraplegin is the product of the *SPG7* gene, mutations in which cause about 4% of recessive familial cases of HSP and up to 12% of sporadic cases [5], [6], [7]. Paraplegin contains an N-terminal mitochondrial targeting sequence (MTS), two transmembrane domains, a AAA (ATPase Associated with various cellular Activities) domain and a C-terminal metal-dependent proteolytic domain. Paraplegin is a subunit of the *m*-AAA (*m*atrix-AAA) protease, a key component of a quality control system that conducts the surveillance of proteins of the inner membrane of mitochondria by degrading in a selective manner non-assembled and damaged polypeptides [8]. Moreover, the *m*-AAA protease mediates proteolytic maturation of specific substrates, such as the mitochondrial ribosomal component MrpL32 [9]. To form a functional *m*-AAA protease, paraplegin assembles with the homologous subunits AFG3L2 or AFG3L1 into a hetero-oligomeric hexameric complex embedded in the inner mitochondrial membrane [10], [11]. In contrast, AFG3L2 and AFG3L1 are also able to form functional homo-oligomers [11]. Notably, two different neurodegenerative diseases, an autosomal dominant form of spinocerebellar ataxia and a recessive early onset spastic-ataxia neuropathy syndrome, have been recently linked to mutations in *AFG3L2* [12], [13]. *AFG3L1* is a pseudogene in human, but it is expressed in the mouse [14].

Several pathogenic mutations, leading to loss-of-function of the protein, have been reported in *SPG7* [5], [6], [7]. To model the disease in the mouse, we previously inactivated the gene by homologous recombination by deleting the first two exons [15]. These exons contain the starting methionine and the mitochondrial targeting sequence. Lack of paraplegin expression was demonstrated in mitochondrial fractions of the knock-out mice. These mice reproduced the HSP phenotype, showing progressive motor impairment from 4.5 months of age, and retrograde axonal degeneration in long descending motor spinal tracts, long ascending sensory spinal tracts, peripheral and optic nerves. Moreover, ultrastructural analyses revealed the early appearance of morphologically abnormal mitochondria in affected axons, alterations that become more pronounced with aging [15]. Intramuscular delivering of *Spg7* cDNA through AAV vectors in knock-out mice stopped the progression of neuropathological changes and rescued the mitochondrial morphology [16].

Here, we report the characterisation of a novel splicing isoform of the murine *Spg7* gene that includes an upstream, alternative first exon. This transcript encodes a novel paraplegin isoform, which we name paraplegin-2 that does not possess the MTS and localizes to the endoplasmic reticulum (ER). The identification of endogenous paraplegin-2 in mouse potentially points to new roles of AAA proteases outside mitochondria and to novel aspects in HSP pathogenesis.

## Results

### An alternative isoform of mouse paraplegin localises to the endoplasmic reticulum

**Figure 1:**
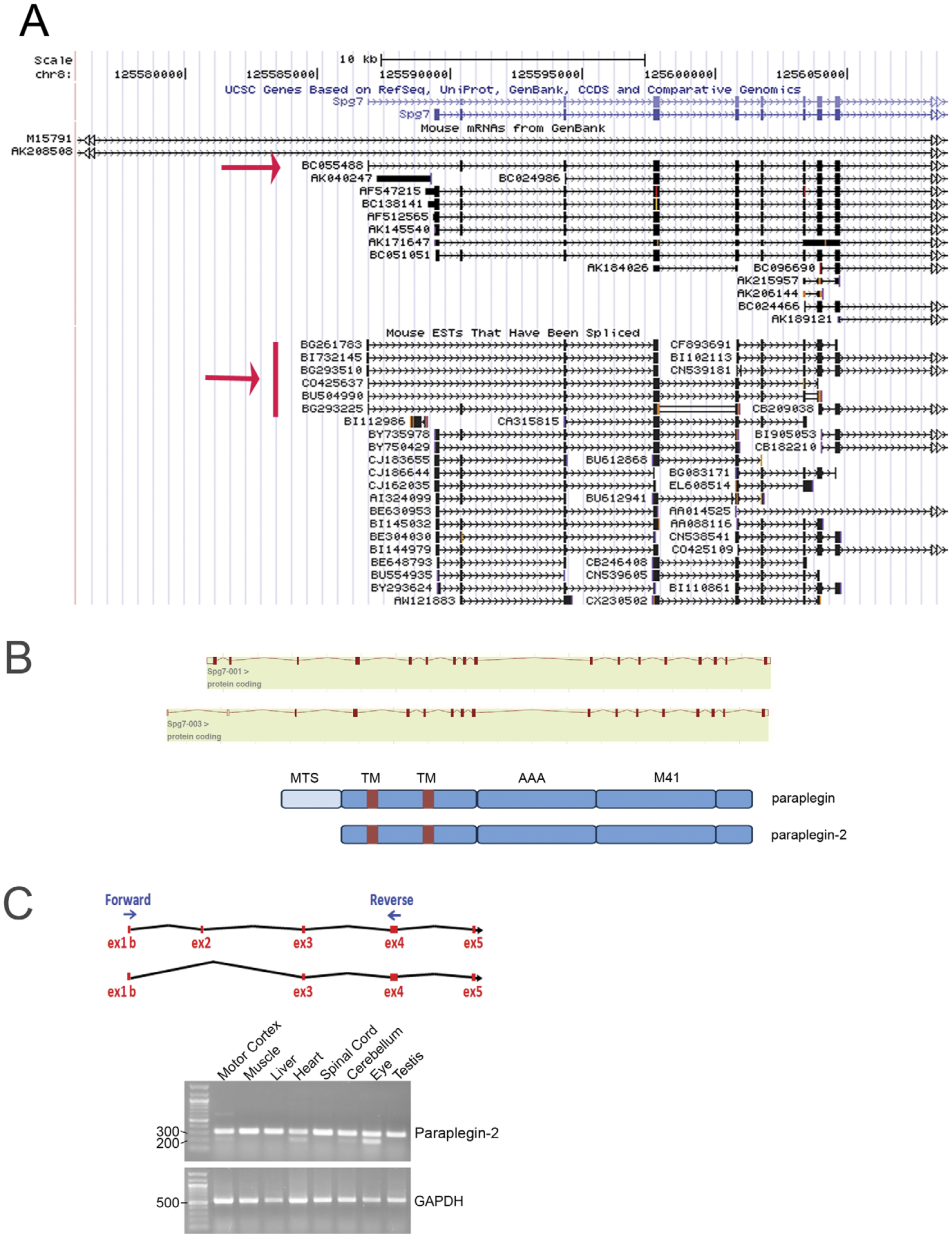
Alternative splicing isoforms of the murine *Spg7* gene. (A) Print-out from UCSC genome browser shows the existence of a complete cDNA (BC055488, red arrow) and six different ESTs (red bar) starting from an upstream alternative exon for the murine *Spg7* gene. These ESTs present either ex1b-2 or ex1b-3 splicing and derives all from eye and retina libraries obtained at different developmental stages. (B) Schematic representation of the two alternative *Spg7* transcripts obtained from the Ensembl database, and of the two respective predicted protein products. MTS, mitochondrial targeting sequence, TM, transmembrane domain, AAA, AAA domain, M41, M41 peptidase domain. (C) RT-PCR for the alternative *Spg7* transcript on several mouse tissue cDNAs. On the top a scheme shows the position of the specific primers. The amplification products are detected as a 210 bp long or a 320 bp long DNA fragments, depending on the splicing pattern. Control amplification was performed using oligonucleotides specific for a housekeeping gene (GAPDH).

Searches of murine ESTs and cDNAs corresponding to murine *Spg7* in public databases identify several clones containing an alternative first exon (exon 1b) located approximately 2.5 kb upstream of the previously described exon 1 (Fig. 1A, B). A complete cDNA (BC055488) and six different ESTs are detected containing exon 1b spliced to either exon 2 or 3 (Fig. 1A). Since exon 1b does not contain any in frame AUG, in both cases the first in frame AUG lies in exon 3, predicting the translation of a shorter paraplegin isoform lacking the first 105 amino acids (dubbed paraplegin-2) (Fig. 1B). Remarkably, all the clones containing exon 1b derive from eye and retina libraries at different developmental stages (Table S1).

We analysed the expression of these alternative splicing isoforms using a RT-PCR approach (Figure 1C). Our results suggest that alternative splicing of *Spg7* comprising exon 1b occurs in all analysed tissues, but the ratio between the splicing ex1b-2 and ex1b-3 presents some tissue variability. Notably the eye shows a strong amplification signal for both the alternative splicing variants.

**Figure 2:**
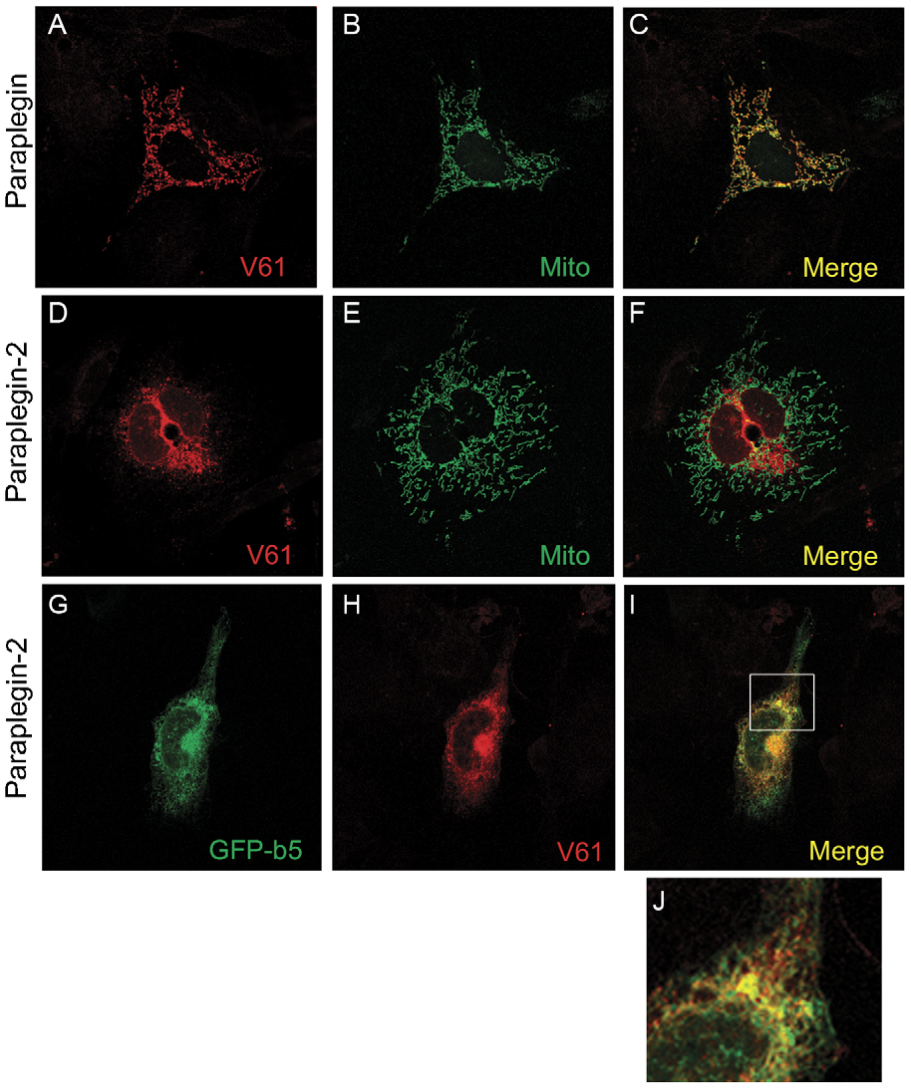
Subcellular localisation of paraplegin-2. Immunofluorescence analysis of paraplegin and paraplegin-2 subcellular localisation in MEF cells after transfection of the respective cDNAs. While paraplegin decorates mitochondria (A–C), paraplegin-2 loses the mitochondrial localisation (D–F), but shows co-localisation with GFP-b5, a markers for the ER (G–I). Panel J shows an enlargement of the box in I. Paraplegin signal is detected using a specific antibody (V61). Mitochondria are labelled by overexpressing a mitochondrially targeted GFP.

Paraplegin is targeted to mitochondria via a MTS encoded by exons 1 and 2. Consistently, prediction of the subcellular localization of paraplegin-2 using the software Mitoprot II returns very low probabilities of mitochondrial targeting. To analyse the subcellular localization of paraplegin-2, we transfected mouse embryonic fibroblasts (MEFs), and NSC34 cells with the BC055488 clone and performed an immunofluorescence assay using a specific α-paraplegin antibody (V61) [15] (Figure 2 and S1). As expected, paraplegin-2 does not target to mitochondria, but assumes a reticular pattern of expression suggesting that it may localise to membranes of the secretory pathway (Fig. 2, D–F). Indeed, we found co-localization between paraplegin-2 signals and ER markers, as an ER-targeted GFP [17], atlastin-1, or seipin (Figure 2, G–I; Figure S1). We conclude that paraplegin-2 localizes to the ER.

### Topology of paraplegin-2 in the endoplasmic reticulum

**Figure 3:**
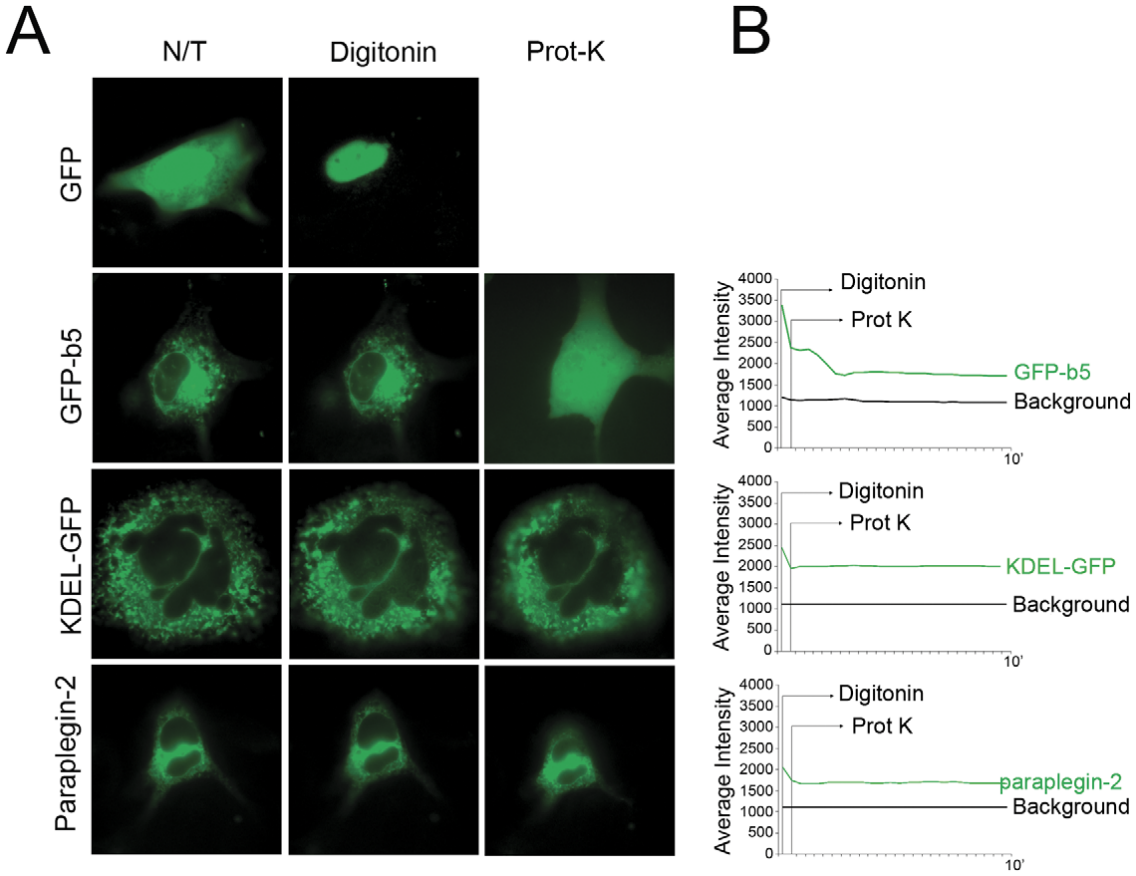
Topology of paraplegin-2. (A) FPP assay on MEFs transfected with different GFP fusion proteins. After permeabilization with digitonin (10 µM) for 1 min, all proteins, with the exception of a cytosolic GFP, retain their localisation in the ER. However, addition of 50 µg/ml proteinase K for 3 min disrupts the fluorescence pattern of cytosolic-exposed GFP-b5, but not that of KDEL-GFP and Paraplegin-2-GFP due to ER membrane protection. (B) Decrease in fluorescence of different GFP-fusion proteins was quantified in live imaging, using a cooled camera driven by METAMORPH software. Images were collected every 5 seconds for 10 minutes. Decrease in signal intensity after addition of proteinase K is visible, as expected, for GFP-b5, but not for KDEL-GFP, and paraplegin-2-GFP.

In mitochondria, paraplegin is inserted via two TM domains in the inner membrane, and exposes its catalytic domains in the mitochondrial matrix. The orientation of integral membrane proteins in the ER is not easily predicted and can depend on the charges of the residues flanking the transmembrane regions. To determine the topology of paraplegin-2 in the ER, we have used a fluorescence protease protection assay (FPP) [18]. This assay uses the restricted proteolytic accessibility of GFP-tagged transmembrane proteins to evaluate their intramembrane orientation. Paraplegin-2-GFP was transfected in MEFs. As controls we also transfected vectors encoding a soluble cytosolic GFP, a KDEL-GFP (retained in the ER), and a GFP fused to the transmembrane domain of cytochrome b5 (GFP-b5, exposed to the cytosolic side of the ER) [17]. 24 hours after transfection, the cells were analyzed using a live-imaging microscope. Digitonin was added at a concentration of 10 µM to selectively permeabilize the cell membrane. As expected, only the signal of freely diffusible cytosolic GFP was lost completely. Cells were then washed with an appropriate buffer followed by proteinase K treatment (50 µg/ml). Proteins contained within a protected cell environment as the lumen of the ER are unaffected by this treatment, while molecules that span the membrane are affected depending on the position of the GFP tag and their topology. We found that paraplegin-2-GFP behaved as the intraluminal KDEL-GFP but unlike GFP-b5, strongly indicating that the C-terminus of the protein, containing the functional domains, is facing the lumen of the ER (Fig. 3).

### Paraplegin-2 is endogenously synthesized in mouse brain and retina

**Figure 4:**
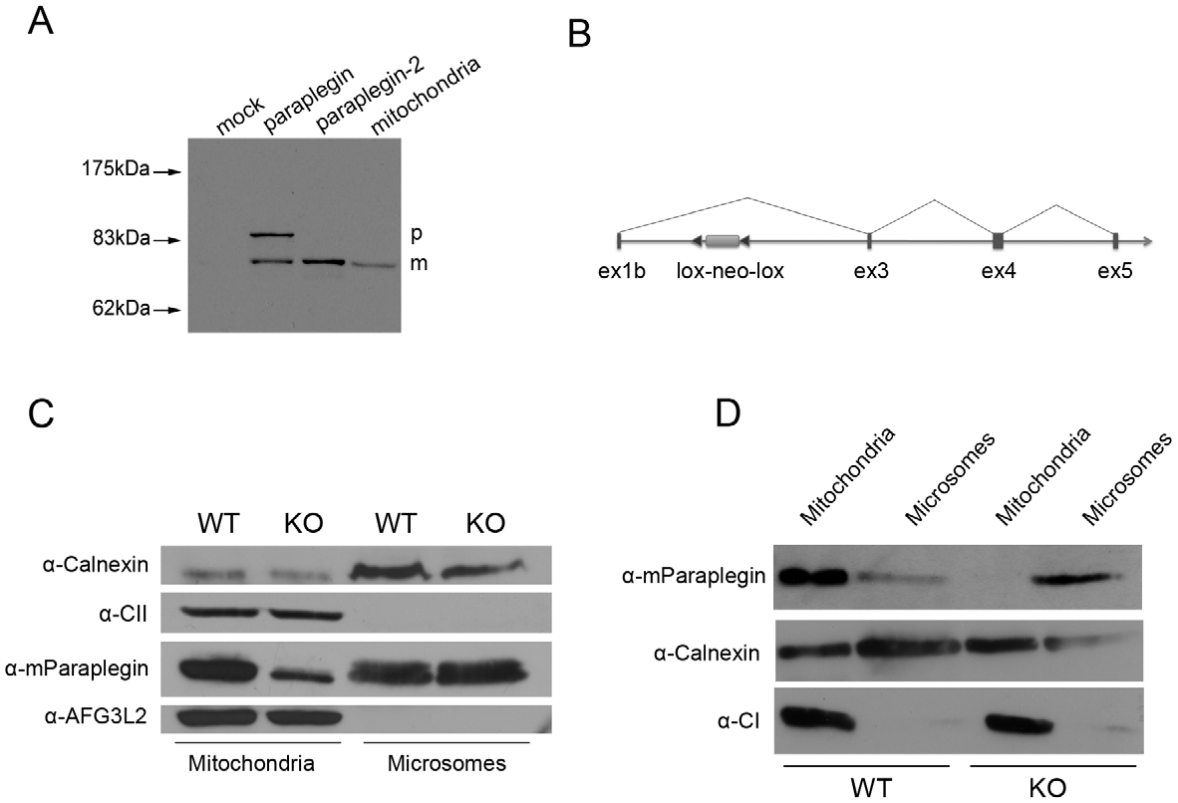
Paraplegin-2 is endogenously expressed in microsomal fractions. (A) Western blot analysis of overexpressed paraplegin or paraplegin-2. Note that paraplegin-2 has the same electrophoretic motility as the mature form of paraplegin. Mitochondrial extracts are loaded as a control. p, precursor; m, mature form. (B) Scheme of the targeted allele in *Spg7* ^−/−^ mice. (C) Western blot analysis of mitochondrial and microsomal fractions obtained from adult control and *Spg7* ^−/−^ mouse brains. Enrichment of mitochondria and microsomes in the fraction were evaluated by using antibodies against 70 kDa subunit of Complex II and calnexin, respectively. A protein with the same molecular weight of paraplegin in the microsomal fractions is recognized by the specific paraplegin antibody. Note that 100 µg of microsomes were loaded versus 30 µg of mitochondria, and that blots were developed using ECL plus to reveal the bands in the microsomes. The paraplegin band in the mitochondrial fraction of *Spg7^−/−^* mice is likely a retrocontamination of mitochondria with microsomes. (D) Western blot analysis of mitochondrial and microsomal fractions obtained from retina of *Spg7^−/−^* and control mice using the specific paraplegin antibody. Pooled retinas from 15 *Spg7* ^−/−^ mice and 12 control mice were used for this experiment. The enrichment of mitochondria and microsomes in the fractions was evaluated by antibodies against NDUFB6 (17 kDa subunit of Complex I) and calnexin, respectively.

An important question is whether paraplegin-2 has any physiological role, and to start to address this issue it is crucial to demonstrate the endogenous existence of the protein. Remarkably, the initial methionine of paraplegin-2 corresponds to the first amino acid of the mature cleaved form of mitochondrial paraplegin [19]. This strongly suggests that this protein may be fully functional in the ER. However, it is impossible to distinguish the two paraplegin isoforms by their electrophoretic motility on a western blot (Fig. 4A).

We previously generated an *Spg7* knock-out mouse model by deleting exons 1 and 2 of the gene [15]. Exon 1b and the upstream promoter region are instead intact in the targeted allele (Fig. 4B). RT-PCR analysis on tissues derived from the knock out mouse showed the presence of the alternative *Spg7* transcript composed of exon1b spliced directly to exon 3 (not shown). Thus our *Spg7* model represents a specific knock out of the mitochondrial paraplegin isoform. Tissues derived from this mouse represent an ideal material to evaluate the endogenous presence of paraplegin-2, since any protein detected can only derive from the alternative transcript. To demonstrate that paraplegin-2 is synthesized endogenously, we purified mitochondrial and microsomal fractions from the brain and retina of wild-type and *Spg7^−/−^* mice. In the brain, a band of the expected molecular weight was detected by the specific paraplegin antibody in both mitochondrial and microsomal fractions in wild-type mice, although paraplegin appeared to be more abundant than paraplegin-2 (Fig. 4C). In *Spg7^−/−^* we detected some residual signal in the mitochondrial fractions, owing to microsome retrocontamination, however the amount of paraplegin detected in the microsomes was the same as in wild-type animals. Notably, we did not detect microsomal signals either for AFG3L2 or for the 70 kDa subunit of Complex II, strongly indicating that this fraction is free from mitochondrial contaminations (Figure 4C).

To further confirm this result, we attempted to purify mitochondrial and microsomal fractions from retina of wild-type and *Spg7* knock-out mice. Remarkably, a band of the correct molecular weight was detected by the V61 antibody in microsomal fractions purified from wild-type and knock-out mice (Fig. 4D). These data strongly indicate that paraplegin-2 is expressed in vivo in both mouse retina and brain, and that this isoform is still present in the *Spg7* knock-out mice.

### Paraplegin-2 requires a molecular partner to form a high molecular weight complex

**Figure 5:**
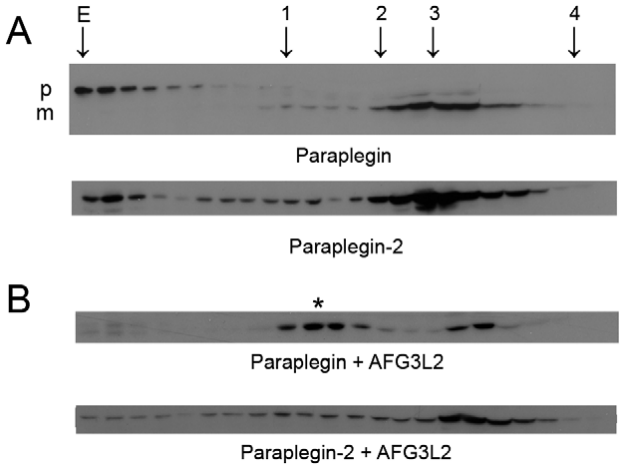
Paraplegin-2 forms high-molecular weight complexes. (A) Gel filtration analysis of paraplegin and paraplegin-2 high molecular weight complexes. Extracts obtained from COS7 cells transfected with paraplegin and paraplegin-2 cDNAs were fractionated by Superose 6 sizing chromatography. Eluate fractions were TCA precipitated and analysed by SDS-PAGE and immunoblotting using V61 α-paraplegin polyclonal antibodies. Overexpression of paraplegin or paraplegin-2 alone does not lead to the formation of a high molecular weight complex. Some aggregates are detected in fractions corresponding to the exclusion volume (E). In the case of paraplegin these aggregates are specific for the uncleaved precursor (p) that accumulates since cleavage of paraplegin depends on the *m*-AAA protease itself [19]. (B) When AFG3L2 is co-expressed, the formation of a high molecular weight complex becomes evident for paraplegin (*), but not for paraplegin-2. Note that paraplegin is now completely cleaved. The following marker proteins were used for calibration: 1, Tyroglobulin (660 kDa); 2, Ferritin (440 kDa); 3, Alcohol Dehydrogenase (150 kDa); 4, Carbonic anhydrase (29 kDa), E indicates the exclusion volume of the column. p, precursor; m, mature form.

AAA proteases must form assemblies to be functional, since the activation of the ATPase domain occurs at the subunit interface [20]. In mitochondria, paraplegin is part of the hexameric *m*-AAA protease. Differently from AFG3L2 and AFG3L1, paraplegin is unable to homo-oligomerize into a functional *m*-AAA protease complex [11]. No isoforms of AFG3L2 and AFG3L1 are known to exist outside mitochondria. We could not identify alternative spliced mRNA for these genes and we excluded that these proteins were present in microsomal preparations (Fig. 3C, and not shown). To exclude that paraplegin-2 is able to homo-oligomerize, we performed a gel filtration assay in COS7 cells overexpressing the protein. As control, we also transfected the mitochondrial isoform. Neither paraplegin nor paraplegin-2 formed high molecular weight complexes when overexpressed alone (Figure 5A), suggesting that the presence or the amount of an interacting partner is limiting in this condition. As expected, co-expression of AFG3L2 with paraplegin, but not with paraplegin-2, induced the formation of a high molecular weight complex (Figure 5B). These data suggest that paraplegin-2 is unable to form a homo-oligomeric complex in the ER without the presence or the right amount of a specific interacting partner.

## Discussion

In this study we characterise paraplegin-2, a novel protein isoform encoded by an alternative splicing of the *Spg7* gene. In contrast to paraplegin, this isoform does not target to mitochondria but to the ER, suggesting that AAA proteases may perform additional functions outside mitochondria.

Several evidences point to a physiological relevance of paraplegin-2. First, various ESTs and a complete cDNA containing exon 1b have been isolated from different mouse libraries. Consistently, we could amplify *Spg7* mRNA containing exon 1b in all mouse tissues we analysed. Second, the first methionine of paraplegin-2 corresponds to the N-terminal residue in mature mitochondrial paraplegin after consecutive cleavage by the matrix metalloprotease and the *m*-AAA protease itself [19], strongly suggesting that the function of paraplegin-2 might be preserved. Finally, we show that paraplegin-2 accumulates endogenously in microsomal fractions purified from mouse brain and retina. All AAA^+^ proteins form functional hexameric complexes after homo-oligomerization or hetero-oligomerization with a highly homologous protein [21], [22]. In murine mitochondria, paraplegin hetero-oligomerizes with AFG3L2 and/or AFG3L1 to form the *m*-AAA protease, but it is unable to homo-oligomerize into a stable complex [11]. Similarly, paraplegin-2 appears unable to form homo-oligomers after overexpression, suggesting that it requires interaction with a molecular partner. Since no evidence for alternative isoforms of AFG3L2 or AFG3L1 targeted to the ER was found, the identity of this potential interactor is still unclear.

These data open up the possibility that AAA proteases might function in the ER. In mitochondria, *m*-AAA proteases play key roles in protein quality control of membrane proteins by degrading misfolded proteins. A similar function could be envisioned for AAA proteases in the ER. The primary mechanism of disposal of misfolded proteins in the ER is the ER-associated degradation (ERAD), which requires delivery of misfolded polypeptides to the 26S proteasome [23]. How soluble ERAD substrates are retrotranslocated to the cytosol is well known, while much less clear is the mechanism by which polytopic, integral membrane substrates are delivered to the ubiquitin-conjugation system and the proteasome [24]. In some cases these substrates are recognized directly by E3 ubiquitin ligases [25], [26], but the presence of additional systems cannot be excluded. Another possibility is that similar to its mitochondrial counterpart, a putative AAA protease in the ER might be implicated in critical biogenesis steps, by promoting the maturation of specific substrates. Future studies will be required to address these issues, by identifying the partner of paraplegin-2 in the complex and possible interactors/substrates. The tissue-specific expression of paraplegin-2 makes this task very challenging. In fact, detection of paraplegin-2 in retina microsomal fraction is in agreement with the origin of all the cDNA clones present in the databases, suggesting that paraplegin-2 might play a tissue-specific role in the retina.

Our data also show that the *Spg7* ^−/−^ mouse should be regarded as an isoform-specific knock-out model, leading to the selective lack of paraplegin from mitochondria. The phenotype of these mice strongly argues for a predominant role of mitochondrial paraplegin in HSP. *Spg7^−/−^* mice recapitulate all the crucial features of the human disease, including the motor deficit and the progressive axonopathy restricted to long central and peripheral axons, and their phenotype could be rescued by reintroduction of the mitochondrial protein [15], [16]. However, an important question elicited by our findings is whether a fraction of paraplegin may exist outside mitochondria also in humans and contribute to some pathogenic aspects of HSP. Evidence for human ESTs containing alternative first exons for *SPG7* can be found in public databases (Fig. S2, Table S2). Again, these transcripts are predicted to encode isoforms of human paraplegin lacking the mitochondrial targeting sequence. Further analyses are needed to confirm these predictions experimentally. Most *SPG7* mutations identified in HSP patients can potentially affect all isoforms, however two pathogenic mutations, one missense (A10S) and one affecting the first methionine, have been found in exon 1 [7], [27], arguing for the fact that it is sufficient to affect mitochondrial paraplegin to induce HSP, similarly to what occur in the mouse. However, a role for an ER localized paraplegin in human disease appears particularly intriguing, since the number of HSP proteins residing in the ER is becoming notable. At least three HSP associated proteins, spastin, atlastin-1, and REEP1 are involved in ER membrane shaping and modelling events and have been shown to interact in the ER membrane [28], [29]. This is an important subgroup, as mutations in the genes encoding these proteins cause up to 60% of HSP cases [28]. Other HSP proteins, as NIPA1 and seipin, also reside in the ER, and when mutated appear to accumulate, and to induce ER stress and subsequent apoptotic death [30], [31]. Future studies are required to address the function of paraplegin-2 and its potential relevance in neurodegeneration.

## Materials and Methods

### DNA constructs

The BC055488 clone was ordered from ImaGenes (IRAKp961N10120Q). Generation of paraplegin-2-GFP construct was obtained by amplification of paraplegin-2 cDNA from BC055488 clone using these oligonucleotides: FW 5′-TTCTCGAGAACACCTCAAGGATGAAGCAG-3′, REV 5′-TTGGATCCCGGGAGCCGGAGCCTCCTC-3′.

The amplified cDNA was then cloned 5′ and in-frame to the GFP cDNA in pEGFP N°2 vector (Clontech). GFP-b5 [32] and KDEL-GFP were kind gift from Nica Borgese, mito-GFP was a kind gift from K. Mihara [33], Atlastin-1-GFP was a kind gift from Andrea Daga.

### RT-PCR

Total RNA was isolated from mouse tissues using TRIzol Kit (Invitrogen) and retro-transcribed into cDNA using SuperScript kit (Invitrogen). Amplification of the alternative splicing isoform of *Spg7* was obtained by RT-PCR using these oligonucleotides: Ex1-b 5′-GAGGAAGCCAGCCACGAGGTG-3′; Ex4 5′-GAGGGAGTTCAGCAGGCTCATG-3′.

For GAPDH amplification we used these oligonucleotides: FW 5′-TGGTGAAGGTCGGTGTGAAC-3′, REV 5′-CAGTGATGGCATGGACTGTG -3′.

### Immunofluorescence analysis and live imaging microscopy

Establishment of MEFs was already described [34]. NSC34 cells were obtained from N. Cashman [35]. Cell lines were cultured in DMEM with 10% Fetalclone III serum (Hyclone) or 5% defined FBS, respectively, and transiently transfected for 24 hours using Lipofectamine2000 (Invitrogen). Immunofluorescence assays were performed as previously reported [36]. Confocal microscopy was performed with a Bio-Rad (Hercules, CA, USA) Radiance 2100 confocal microscope at 1024×1024-pixel resolution. To perform live imaging FPP assay, cultures were transferred to a live cell imaging workstation composed of an inverted microscope (Nikon Eclipse TE2000-S+BD CARV II, Nikon, Melville, NY, USA), a heated (37°C) chamber and a Nikon Plan Apo VC 100X/1.40 oil objective. Cells were incubated with digitonin 10 µM for 1 min and followed by addition of proteinase K 50 µg/ml. Images were collected every 5 seconds for 10 min using a cooled camera (QUANTEM 512 SC) driven by METAMORPH software (Molecular Devices Ltd, Downingtown, PA, USA). The same software was used for fluorescence quantification measurements.

### Subcellular fractionation and gel filtration assay

All animal procedures were conducted in accordance with European (EU directive 86/609/EEC), national (TierSchG), and institutional guidelines and protocols, and were approved by local governmental authorities (Landesamt für Natur, Umwelt und Verbraucherschutz Nordrhein-Westfalen) under the license 87–51.04.2010.A219.

For mitochondria and microsomes purification, mouse brains and retinas were homogenized (10 strokes) in 10 volumes/g of wet tissue isolation buffer (220 mM D-Mannitol, 70 mM Sucrose, 20 mM Hepes, 1 mM EDTA and 0.1% w/v BSA, pH = 7.2) using a glass/Teflon pestle. The crude homogenate was sequentially centrifuged at 500 g and 1,000 g for 10 min and then the supernatant was centrifuged again at 8,000 g for 10 min for mitochondria precipitation. The crude mitochondrial fraction was resuspended in 6 volumes/g of wet tissue of isolation buffer and purified again with sequential centrifugation steps of 10 min each at 500 g, 1,000 g and 8,000 g. The supernatant fraction was instead cleaned by two centrifugations at 20,000 g for 10 min, before performing microsomal precipitation at 150,000 g for 2 hours. Both mitochondrial and microsomal fractions were resuspended in RIPA buffer (50 mM Tris-HCl, 1% w/v NP-40, 0.25% w/v Sodium Deoxicolate, 150 mM NaCl and 1 mM EDTA, pH = 7.4) for SDS-PAGE experiments and in GF buffer (30 mM Tris-HCl pH = 7.4, 10 mM Mg-acetate, 150 mM K-acetate pH = 7.4, 1 mM PMSF and 5 mM ATP) for gel filtration assays.

Gel filtration analyses were performed as previously reported [11].

### Antibodies

Specific rabbit polyclonal antibodies against mouse paraplegin and AFG3L2 were previously described [15], [34]. Mouse monoclonal antibodies against α-70 kDa subunit of Complex II and anti- NDUFB6 (17 kDa subunit of Complex I) were purchased from Invitrogen; rabbit polyclonal anti-calnexin antibody was purchased from Stressgen.

## Supporting Information

Figure S1 **Paraplegin-2 localises to the endoplasmic reticulum in NSC34 cells.** Immunofluorescence analysis of paraplegin-2 subcellular localisation in NSC34 cells after co-transfection with constructs encoding mCherry-seipin or atlastin1-myc-GFP. The latter proteins are known to localise to the endoplasmic reticulum. Paraplegin signal is detected using a specific antibody (V61).
(TIF)

Figure S2 **Putative human alternative** ***SPG7*** **ESTs.** Schematic view of the human *SPG7* transcripts and of two ESTs containing putative alternative first exons (only the first exons are shown).
(TIF)

Table S1 Murine *Spg7* cDNAs and ESTs containing exon1b.
(DOCX)

Table S2 Human *SPG7* ESTs containing alternative first exons.
(DOCX)
