# Supplementary figures and images for: Alternative Splicing of Spg7, a Gene Involved in Hereditary Spastic Paraplegia, Encodes a Variant of Paraplegin Targeted to the Endoplasmic Reticulum
Source: PLoS One. 2012 May 1;7(5):e36337. doi: 10.1371/journal.pone.0036337 (PMC3341365; doi:10.1371/journal.pone.0036337)

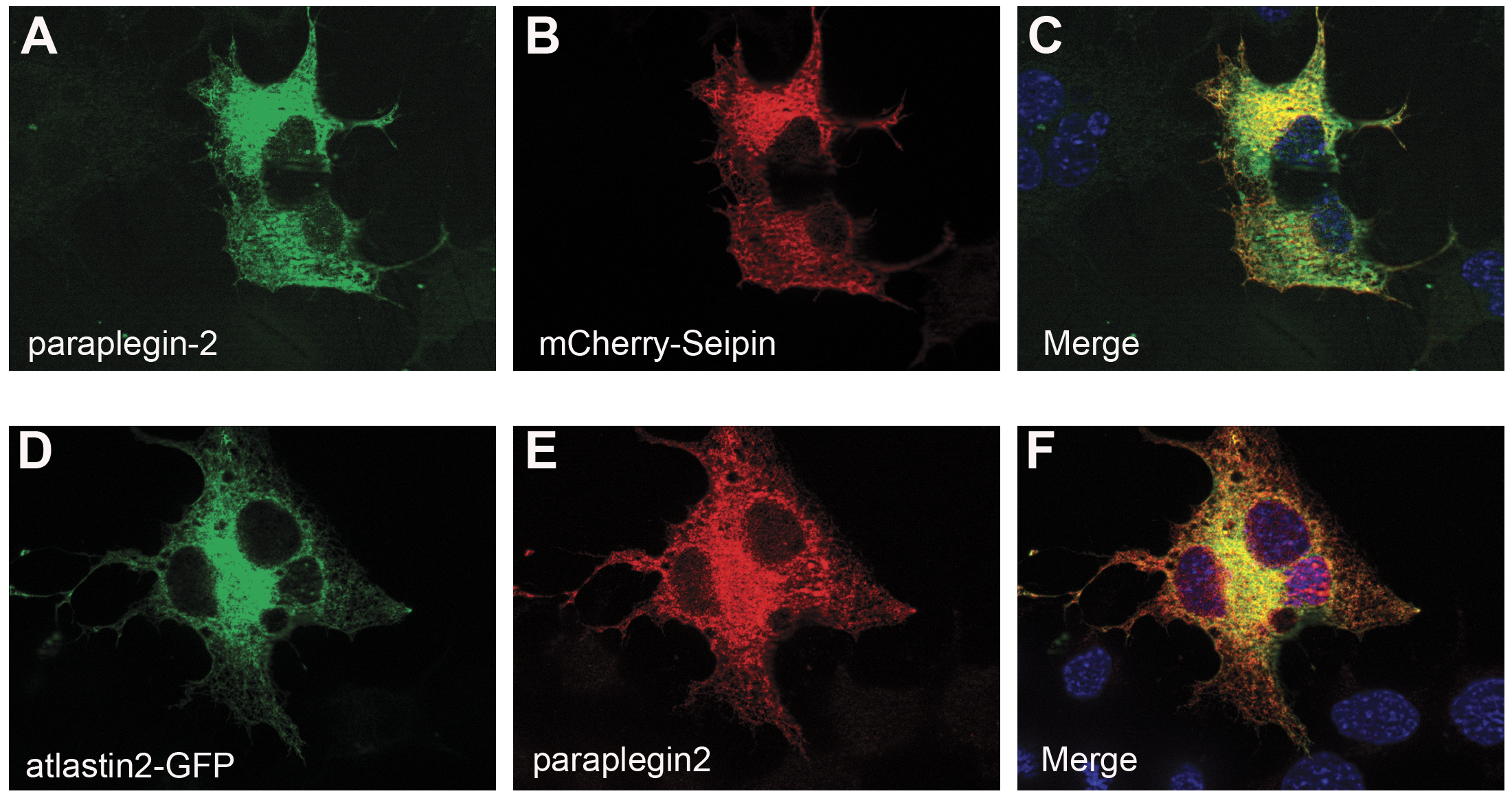

Supplement: Figure S1 — Paraplegin-2 localises to the endoplasmic reticulum in NSC34 cells. Immunofluorescence analysis of paraplegin-2 subcellular localisation in NSC34 cells after co-transfection with constructs encoding mCherry-seipin or atlastin1-myc-GFP. The latter proteins are known to localise to the endoplasmic reticulum. Paraplegin signal is detected using a specific antibody (V61). (TIF) [file pone.0036337.s001.tif]

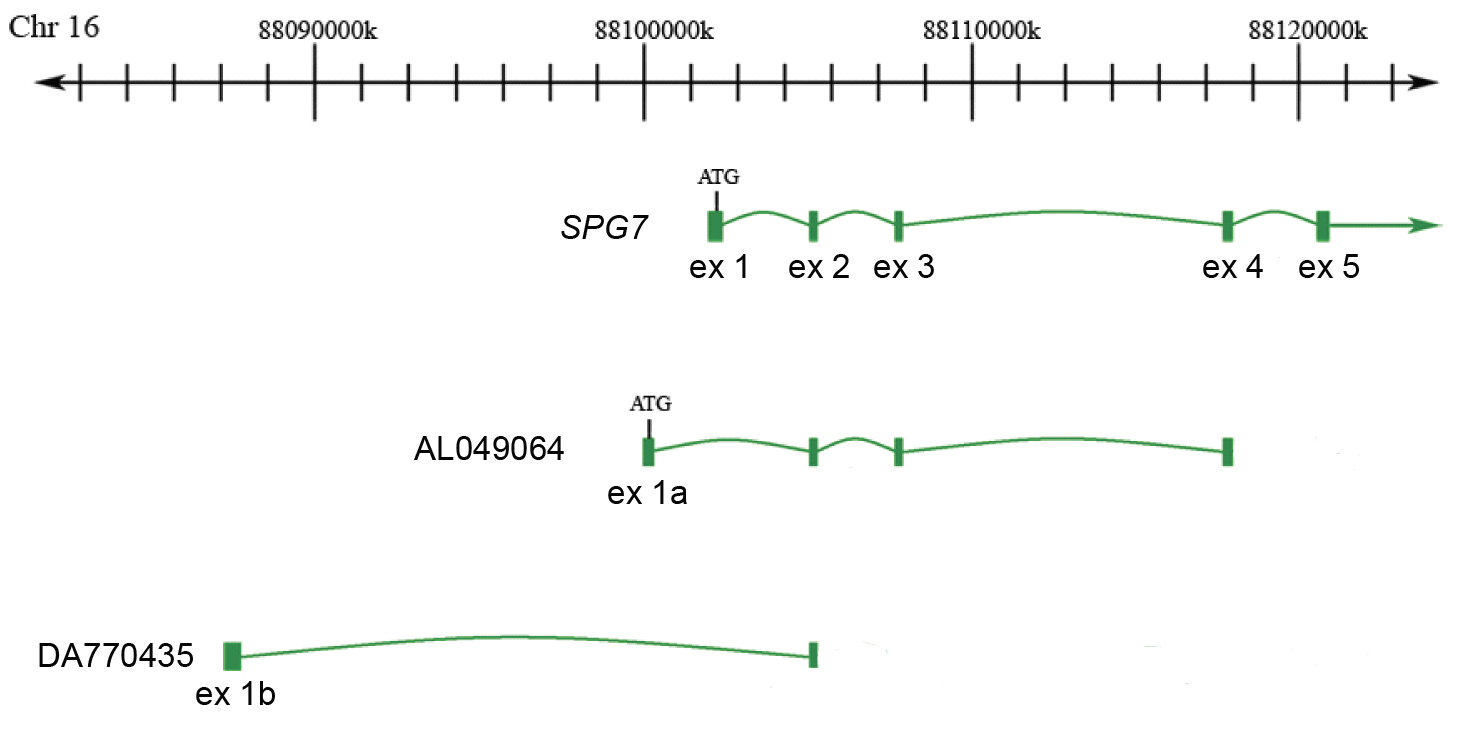

Supplement: Figure S2 — Putative human alternative SPG7 ESTs. Schematic view of the human SPG7 transcripts and of two ESTs containing putative alternative first exons (only the first exons are shown). (TIF) [file pone.0036337.s002.tif]
