# Supplementary material for: Alternative Splicing of Spg7, a Gene Involved in Hereditary Spastic Paraplegia, Encodes a Variant of Paraplegin Targeted to the Endoplasmic Reticulum
Source: PLoS One. 2012 May 1;7(5):e36337. doi: 10.1371/journal.pone.0036337 (PMC3341365; doi:10.1371/journal.pone.0036337)
Supplement: Table S1 — Murine Spg7 cDNAs and ESTs containing exon1b. (DOCX) [file pone.0036337.s003.docx]

**Table S1. Murine *Spg7* cDNAs and ESTs containing exon1b**

| Clone | Type | Tissue | Splicing | First in frame ATG |
| --- | --- | --- | --- | --- |
| BC055488 | mRNA | Retina/Eye | 1b-2 | Exon 3 |
| BG261783 | EST | Retina | 1b-3 | Exon 3 |
| BI732145 | EST | Retina | 1b-3 | Exon 3 |
| BG293510 | EST | Retina | 1b-3 | Exon 3 |
| CO425637 | EST | Eye | 1b-3 | Exon 3 |
| BU504990 | EST | Retina | 1b-3 | Exon 3 |
| BG2932225 | EST | Retina | 1b-3 | Exon3 |
