# Supplementary material for: Alternative Splicing of Spg7, a Gene Involved in Hereditary Spastic Paraplegia, Encodes a Variant of Paraplegin Targeted to the Endoplasmic Reticulum
Source: PLoS One. 2012 May 1;7(5):e36337. doi: 10.1371/journal.pone.0036337 (PMC3341365; doi:10.1371/journal.pone.0036337)
Supplement: Table S2 — Human SPG7 ESTs containing alternative first exons. (DOCX) [file pone.0036337.s004.docx]

**Table S2. Human *SPG7* ESTs containing alternative first exons.**

| Clone | Type | Tissue | Splicing | First in frame ATG |
| --- | --- | --- | --- | --- |
| DA770435 | EST | Fetal brain | 1b-2 | Exon 4 |
| AL049064 | EST | Adult testis | 1a-2 | Exon 1a |
